# The influence of esmolol on right ventricular function in early experimental endotoxic shock

**DOI:** 10.14814/phy2.13882

**Published:** 2018-10-14

**Authors:** Lex M. van Loon, Johannes G. van der Hoeven, Peter H. Veltink, Joris Lemson

**Affiliations:** ^1^ Biomedical Signals and Systems Faculty of Electrical Engineering, Mathematics and Computer Science Technical Medical Centre University of Twente Enschede the Netherlands; ^2^ Department of Critical Care Medicine (707) Radboud university medical center Nijmegen the Netherlands

**Keywords:** Beta‐blocker esmolol, microcirculation, right ventricular function, Sepsis, ventricular‐arterial coupling

## Abstract

The mechanism by which heart rate (HR) control with esmolol improves hemodynamics during septic shock remains unclear. Improved right ventricular (RV) function, thereby reducing venous congestion, may play a role. We assessed the effect of HR control with esmolol during sepsis on RV function, macrocirculation, microcirculation, end‐organ‐perfusion, and ventricular‐arterial coupling. Sepsis was induced in 10 healthy anesthetized and mechanically ventilated sheep by continuous IV administration of lipopolysaccharide (LPS). Esmolol was infused after successful resuscitation of the septic shock, to reduce HR and stopped 30‐min after reaching targeted HR reduction of 30%. Venous and arterial blood gases were sampled and the small intestines’ microcirculation was assessed by using a hand‐held video microscope (CytoCam‐IDF). Arterial and venous pressures, and cardiac output (CO) were recorded continuously. An intraventricular micromanometer was used to assess the RV function. Ventricular–arterial coupling ratio (VACR) was estimated by catheterization‐derived single beat estimation. The targeted HR reduction of >30% by esmolol infusion, after controlled resuscitation of the LPS induced septic shock, led to a deteriorated RV‐function and macrocirculation, while the microcirculation remained depressed. Esmolol improved VACR by decreasing the RV end‐systolic pressure. Stopping esmolol showed the reversibility of these effects on the RV and the macrocirculation. In this animal model of acute severe endotoxic septic shock, early administration of esmolol decreased RV‐function resulting in venous congestion and an unimproved poor microcirculation despite improved cardiac mechanical efficiency.

## Introduction

Sepsis is one of the leading causes of death among hospitalized patients and a frequent cause of admission to intensive care units (ICUs)(Mayr et al. 2014). Morbidity and mortality from sepsis in ICU patients remain high, despite an improved understanding of the pathophysiology of sepsis (Angus et al. 2001; Martin et al. 2003). Therefore, the search for effective therapeutic strategies remains relevant (Ince 2015).

Heart rate (HR) control by esmolol, a an ultrashort acting β1‐adrenoceptor antagonist could be such a novel therapeutic strategy with substantial impact on the clinical management of septic shock. Reduced HR could improve the cardiac mismatch of oxygen demand and supply in septic patients, by decreasing myocardial oxygen consumption (Hosokawa et al. 2017). The study by Morelli et al. shows that esmolol is able to control noncompensatory tachycardia during established septic shock after 24 h of hemodynamic optimization and reduce mortality from 81% to 49%. Together with a reduction in HR, they showed that esmolol was able to improve macrocirculatory variables significantly, while the microvascular blood flow was preserved and the frequency of adverse events did not increase (Morelli et al. 2013a).

Although these results look promising, the optimal dosage, initiation and the mechanism by which esmolol might improve hemodynamic and organ function and reduce mortality remain to be elucidated (Pinsky 2013; Orbegozo Cortes et al. 2014). It is hypothesized that HR reduction during septic shock may improve right ventricular (RV) diastolic function, leading to increased cardiac preload by reducing venous congestion and thereby increasing effective organ perfusion (Harkin et al. 2000; Park et al. 2008; Legrand et al. 2013; Morelli et al. 2013b; Ince 2015). These possible positive effects of esmolol on RV function have only been described using echocardiography (Morelli et al. 2014). However, to obtain more mechanistic insights, a comprehensive approach using intraventricular measurements should be performed. Another supposed beneficial effect of esmolol in septic patients may be related, at least in part, to reduced afterload by better ventricular–arterial (V–A) coupling. V‐A coupling is a key determinant of cardiovascular function by describing the interaction between the ventricle (contractility) and the arterial system (afterload)(Razzolini et al. 2004; Dekleva et al. 2015; Morelli et al. 2016). Decoupling of contractility and afterload is clearly present in patients with septic shock and may persist after resuscitation to recommended hemodynamic targets (Guarracino et al. 2013, 2014).

In this study, we aimed to elucidate the effect of early initiation of HR control by esmolol on RV function in the acute stage of resuscitated sepsis (<24 h). Using a lamb model of resuscitated severe septic shock, we investigated both the RV function and V‐A coupling, the macrocirculation, microcirculation and end‐organ‐perfusion as a result of esmolol infusion.

## Materials and Methods

This experiment was performed after approval of the local ethics committee on animal research of the Radboud University Medical Center (RUMC License number RU‐DEC 2014–10) and in full compliance with Dutch and European legal requirements on the use and protection of laboratory animals. Ten conventionally reared female lambs (crossbred Texelaar‐Flevolanders) under general anesthesia were studied. No control group was considered necessary since the animals acted as their own control.

### Anesthesia and ventilation

Premedication consisted of midazolam (0.5 mg/kg) and ketamine (4 mg/kg), anesthesia was performed using IV administration of propofol (2 mg/kg). After endotracheal intubation, general anesthesia was maintained using inhalation of isoflurane (0.5–2 volume%), the continuous IV administration of sufentanil (2 *μ*g/kg per h) and rocuronium (1 mg/kg per h) after a loading dose of 1 mg/kg. The lungs were mechanically ventilated using a volume‐controlled mode with tidal volumes of 8–10 mL/kg, 5 cmH_2_O PEEP, FiO_2_ 0.4, and an inspiratory‐to‐expiratory ratio of 1:2. Ventilation was adjusted according to the end tidal CO_2_ level. Impaired oxygenation was treated by increasing PEEP and or increasing the FiO_2_ to maintain an oxygen saturation > 95%. During the experiment, continuous IV dextrose 10% 2.4 mL/kg per h (240 *μ*g/kg per min dextrose) was administered supplemented with IV 0.9% saline 2–4 mL/kg per h in order to maintain fluid balance and to prevent hypoglycemia. At the end of the experiment, the animals were euthanized with an overdose of pentobarbital (150 mg/kg IV) (T3).

### Surgical preparation

All lambs were positioned in dorsal position for inserting the intravascular catheters using surgical cut down procedures. A 7.5F x 20 cm triple‐lumen central venous catheter was placed in the left internal jugular vein (Multicath, Vygon Nederland BV) for central venous blood pressure monitoring, venous blood sampling, and for the administration of fluid and drugs. A 18G × 10 cm single‐lumen catheter (Leaderflex, Vygon Nederland BV) was introduced in the right femoral artery for arterial blood pressure monitoring and blood sampling.

A 5F × 8 cm introducer sheath (Cordis Europe, Waterloo, Belgium) was introduced followed by a 5F guiding catheter (Impulse™, Boston scientific, Kerkrade, The Netherlands) into the right internal jugular vein. The guiding catheter was advanced using fluoroscopy until RV blood pressure was recorded at the tip. The ComboWire^®^ XT (Volcano Corporation, Rancho Cordova, CA), a 0.014‐in dual‐sensor (pressure and Doppler velocity)‐equipped guide wire, was advanced into the guiding catheter. The guiding catheter was pulled back in order to locate its tip in the right atrium and leave the pressure wire in the RV. The ComboWire^®^ XT pressure wire was used to perform RV pressure measurements continuously.

Next, the lambs were repositioned for the duration of the experiment in the right lateral position. An ultrasound transit‐time perivascular flow probe (14 or 16 mm) (PAX series, Transonic Systems, Ithaca, NY) was placed around the main pulmonary artery to measure CO after left thoracotomy. Laparotomy created a window for Incident Dark Field (IDF) imaging the of the small intestines’ microcirculation. Gastrostomy was performed in order to place a 30 cm tube for gastric decompression. Last, an 8F silicon Foley catheter (Covidien, Mansfield, MA 02048, USA) was introduced into the bladder.

### Resuscitated endotoxic shock

**Figure 1:**
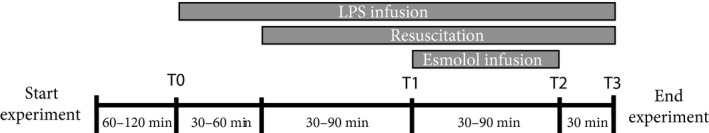
Schematic overview of the experimental design.

After closing all the incisions, the chest and the abdomen, and prior to creating a situation of resuscitated endotoxic shock, we gave a fluid bolus (5–10 mL/kg) to treat potential initial hypovolemia related to induction of anesthesia and surgical manipulation. Next, a stabilization period of 30 min (T0) was followed by continuous IV administration of lipopolysaccharide (3 *μ*g/kg per h) (LPS, US Standard Reference Endotoxin *Escherichia coli* O:113) after a loading dose of 3 *μ*g/kg, see Figure 1. The LPS dose was defined from previous pilot experiments. Resuscitation started 30 min after LPS induced a 50% reduction in CO or a 25% reduction in ABP (T1). Resuscitation objectives were to return ABP and CO to their baseline values. A fluid load of 50 mL of saline (or whole blood if hemoglobin level<5 mmol/L) was administered initially to evaluate fluid responsiveness guided by flow probe CO; in case of absence of fluid response, nor‐epinephrine and/or dobutamine were used. The initial dose of nor‐epinephrine and dobutamine was 0.1 *μ*g/min per kg and 0.5 *μ*g/min per kg, respectively, and could be increased to up to 1.5 *μ*g/min per kg and 2.5 *μ*g/min per kg, respectively to achieve resuscitation goals. In all animals this protocol leads to restoration of blood pressure and CO.

### Experimental protocol

Thirty minutes after creating a situation of resuscitated endotoxic shock (T1), esmolol (Baxter,Maurepas, France) was started at 50 *μ*g/kg per min and progressively increased to reduce in the HR by 30%. A percentage change – in the same magnitude as Morelli et al. (2013a)‐ was used to allow for differences in resting HR. Resuscitation maneuvers were maintained or increased in order to maintain ABP and CO at baseline values; except for dobutamine dosage, which was kept constant in order not to intervene with the esmolol treatment. Esmolol infusion was stopped 30 min after the HR reached the targeted reduction of 30% (T2), see Figure 1.

### Data collection

Electrocardiography (EKG), blood pressures, and pulmonary flow were simultaneously and continuously recorded on a laptop computer and stored on a hard disk with a sample rate of 200 Hz by an A/D converter (NI USB‐6211, National Instrument, Austin, TX, USA). Before, during, and after the esmolol infusion, five sequences of 10s clips of the small intestines’ microcirculation were obtained with the Cytocam‐IDF video microscope (Braedius Medical, Huizen, the Netherlands). Image acquisition was performed according to published consensus criteria (De Backer et al. 2007). A hand‐held iSTAT® point‐of‐care analyzer (Abbott Laboratories, IL, USA) was used to obtain arterial hematocrit, hemoglobin, partial pressure of CO2 and Urea.

### Data analysis

A blinded investigator (LvL) scored the captured IDF clips according to Massey et al. In short, images were scored on six categories: illumination, duration, focus, content, stability, and pressure. Videos are assigned a score of 0 = good, 1 = acceptable, or 10 = unacceptable for each category. Any video with composed scored ≥10 was discarded from future analysis. Automated Vascular Analysis (AVA 5; Microvision Medical B.V.) was used for off‐line analysis on the images of the sublingual region that met the quality requirements. The continuously recorded EKG, pressure and flow signals were analyzed using custom‐written MATLAB scripts (Matlab R2017b, The MathWorks Inc. Massachusetts, USA). Mean ABP, CVP and CO were acquired by taking a fourth order Butterworth low‐pass filter with a cuff‐off frequency of 0.02 Hz from their raw signal. HR was acquired by automatic detection of R‐peaks from the EKG‐signal.

RV contractile function (dP/dt_max/P) was acquired by taking the peak‐first‐derivative of RV pressure waveform divided by the pressure at that point. The maximum isovolumetric pressure (P_max_) provides an estimate of the maximum pressure that could be generated during an isovolumetric contraction and was determined by fitting a sinusoid to the isovolumetric regions of the pressure tracing, the so‐called “single beat method” (Sunagawa et al. 1980; Takeuchi et al. 1991; Lambermont et al. 2004). Tau was calculated by the method of Weiss et. al, assuming a zero asymptote (Weiss et al. 1976). Ventricular‐arterial coupling ratio (VACR) of the RV was approximated by dividing end‐systolic pressure (Pes) by the difference between P_max_ and Pes (Truong et al. 2015).

### Statistical analysis

Prism Statistical Software was used for statistical analysis (Graph‐Pad Prism 5, GraphPad Software Inc., San Diego, CA, USA). RV systolic and diastolic parameters were normalized to baseline. One‐way analysis of variance (ANOVA) and the Bonferroni test was used for multiple post‐hoc comparisons of the different time points. A *P*‐value of < 0.05 was considered to indicate significance (*=*P* < 0.05, **=*P* < 0.01, and ***=*P* < 0.001).

## Results

A total of 10 female lambs were studied. They were 6‐8‐month‐old, weighing a mean of 20.6 kg [range: 13–24.5 kg] and with a mean body surface area of 0.91 m^2^ [range: 0.67–1.0 m^2^]. All animals included in the study were considered healthy on physical examination when entering the animals’ laboratory. In all animals, LPS infusion plus resuscitation maneuvers caused a septic shock with increased CO, tachycardia, and significantly decreased microcirculatory parameters. Lactate increased significantly over the course of the experiment and was produced aerobic (Pv‐aCO_2_/Ca‐vO_2_ < 1.4 and ScvO_2_ > 70%) (Table 1). Hematocrit and hemoglobin remained within physiological (baseline) ranges.

**Table 1:** Effect of esmolol on hemodynamic and metabolic parameters. Data are expressed as mean ± SD

Variable	Baseline (T0)(*n* = 10)	Resuscitated endotoxic shock (T1) (*n* = 10)	Esmolol (T2) (*n* = 10)	Stop (T3) (*n* = 8)	Sign (T0–T1)	Sign (T1–T2)	Sign (T2–T3)	*P* a
Systolic arterial pressure (mmHg)	76 ± 11	74 ± 11	46 ± 12	67 ± 11	NS	***	**	*P* < 0.001
Diastolic arterial pressure (mmHg)	49 ± 8	48 ± 8	32 ± 8	43 ± 9	NS	***	NS	*P* < 0.001
RV dP/dt_min (mmHg/sec)	−1.3 ± 0.7	−1.8 ± 0.5	−1.4 ± 0.4	−1.6 ± 0.5	NS	NS	NS	NS
Cardiac index (L/min per m^2^)	3.5 ± 1.1	4.5 ± 1.5	2.4 ± 0.8	3.3 ± 0.6	NS	**	NS	*P* < 0.01
Lactate (g/mol)	1.9 ± 1.3	3.5 ± 0.9	4.3 ± 1.2	4.3 ± 1.2	*	NS	NS	*P* < 0.001
Hemoglobin (g/dL)	4.6 ± 0.6	5.5 ± 0.7	5.3 ± 0.8	5.4 ± 0.7	*	NS	NS	*P* < 0.05
Hematocrit (L/L)	0.22 ± 0	0.26 ± 0	0.25 ± 0	0.25 ± 0	*	NS	NS	*P* < 0.05
Urea (mmol/L)	7.1 ± 1.3	7.5 ± 1.5	8.2 ± 2.3	7.5 ± 1.2	NS	NS	NS	NS
PaCO_2_ (mmHg)	43 ± 5.8	49 ± 12.6	45 ± 4.8	47 ± 2.3	NS	NS	NS	NS
PvCO_2_ (mmHg)	46 ± 3.9	53 ± 13.4	51 ± 5.2	51 ± 1.5	NS	NS	NS	NS
ScvO_2_ (%)	86 ± 4	90 ± 4	87 ± 5	89 ± 4	NS	NS	NS	NS
pH	7.4 ± 0.06	7.3 ± 0.09	7.3 ± 0.5	7.3 ± 0.08	*	NS	NS	*P* < 0.05
Base excess (mmol/L)	3.4 ± 3.7	−0.4 ± 3.9	0 ± 4.4	0 ± 6.6	NS	NS	NS	NS
HCO3 (mmol/L)	27.8 ± 3.1	24.7 ± 2.2	24.5 ± 2.0	24.3 ± 2.0	NS	NS	NS	*P* < 0.05

NS, Not significant.

a Significant changes over time (comparison made using one‐way ANOVA), *= *P* < 0.05, **= *P* < 0.01 and ***= *P* < 0.001.

### Effect of esmolol

**Figure 2:**
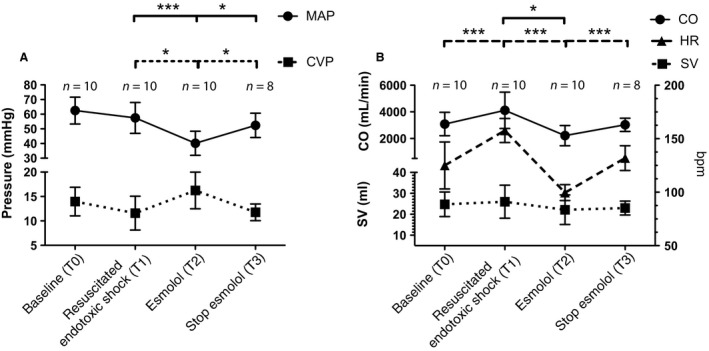
Macrocirculatory variables per study phase. Data are expressed as mean ± SD. Bonferroni's post‐hoc test was used to perform pairwise comparisons between phases. *=*P*  < 0.05, and, ***=*P* < 0.001. (A) Mean arterial pressure (MAP) and central venous pressure (CVP). (B) Cardiac output (CO), Heart rate (HR) and stroke volume (SV).

**Figure 3:**
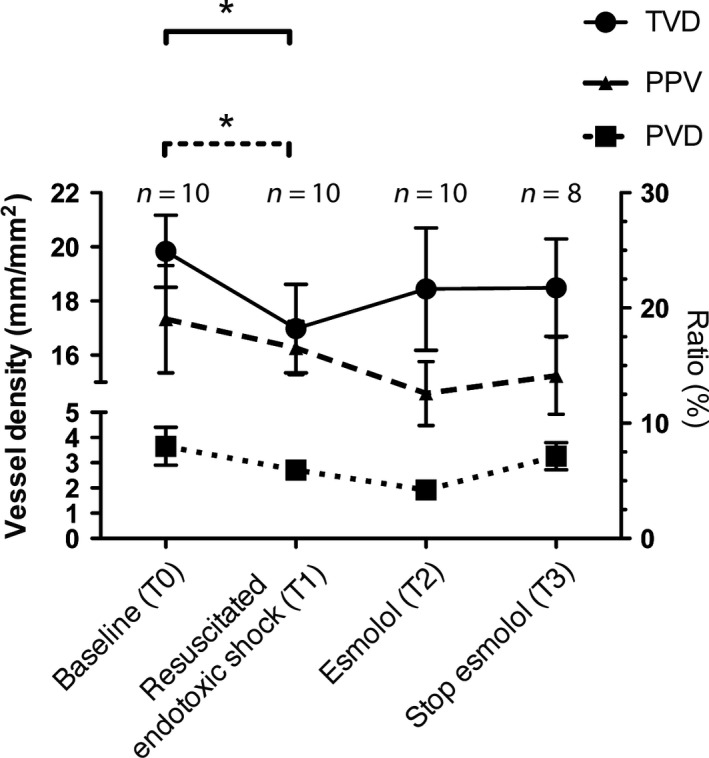
Microcirculatory variables per study phase. Data are expressed as mean ± SD. Bonferroni's post‐hoc test was used to perform pairwise comparisons between phases, *=*P* < 0.05. Microcirculatory parameters: total vessel density (TVD), proportion of perfused vessels (PPV), and perfused vessel density (PVD).

Esmolol infusion induced on average a HR reduction of 37% [range: 31–41%]. This was accompanied with a significant impairment in MAP, CI, and both RV function parameters (systolic and diastolic), see Figure 2 and Table 1. This impairment led to venous congestion, indicated by a significantly increased CVP. The microcirculation of the small intestines remained depressed ultimately, see Figure 3. Apart from the microcirculation, these effects were reversible by stopping esmolol infusion, showing that the deteriorated hemodynamics was not due to progressive endotoxic effects of LPS.

**Figure 4:**
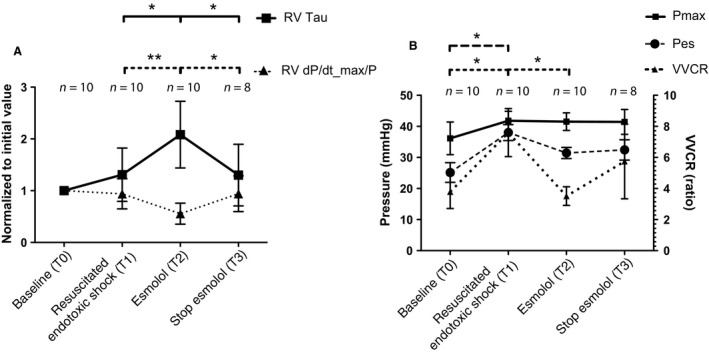
Right ventricular variables per study phase. Data are expressed as mean ± SD. Bonferroni's post‐hoc test was used to perform pairwise comparisons between phases, *=*P* < 0.05, and **=*P* < 0.01. (A) Right ventricular diastolic relaxation time constant (Tau) and right ventricular systolic function (dP/dt_max/P). (B) Right ventricular maximum isovolumetric pressure (P_max_), ventricular‐vascular coupling ratio (VACR), and right ventricular end‐systolic pressure (Pes).

The measurements of Pes, P_max_, and VACR by single beat estimation are shown in Figure 4B. During esmolol infusion, a trend toward a decreased RV end‐systolic pressure and increased estimated RV maximum pressure was seen. This trend resulted in a significant difference between both, showing that esmolol forced the RV to operate below its maximum capacity. This was accompanied by a significant decrease in the VACR (from 7.6 to 3.5 on average), indicating increased cardiac mechanical efficiency during the infusion of esmolol.

### Effect of esmolol on resuscitation requirements

Vasopressor requirements increased during esmolol infusion, while only an additional 2 mL/kg saline [range: 0–5.6 mL/kg] was given in boluses guided by the continuous CO monitoring. This limited amount of fluid showed the reduced fluid responsiveness of the animals during esmolol infusion. Maximum nor‐epinephrine dosage increased from 1.4 *μ*g/min per kg at T1 to 1.8 *μ*g/min per kg at T2 [range: 1–2.7 *μ*g/min per kg], and returned to 1.5 *μ*g/min per kg at T3. Dobutamine administration remained unchanged, as stated in the protocol. Two animals became severely bradycardic during esmolol infusion necessitating two boluses of epinephrine and CPR. Therefore, we excluded their data from phase after stopping esmolol.

## Discussion

In this experimental model of acute septic shock, beta‐blockade with esmolol decreased HR with 37% and induced a significant worsening in macrohemodynamic parameters without recovery of the microcirculation. We showed that the negative effects of esmolol on the macrocirculation could be attributed to a decreased RV function.

We created a model of resuscitated septic shock to test the efficacy of beta‐blockade using esmolol. The severity of initial shock was characterized by increased lactate levels in combination with a significant reduction in CO, MAP, and SV in all animals. Resuscitation with fluids and vasoactive drugs had a positive effect on all macrohemodynamic parameters and changed a hypodynamic hypotensive “cold” shock, induced by LPS, into a “warm” (or hyperdynamic) septic shock. In doing so, we feel that this model was robust enough to resemble clinical significant severe septic shock in humans.

In contrast to other animal and human studies (Suzuki et al. 2005; Mori et al. 2011; Morelli et al. 2014; Hernández et al. 2016), our results revealed that esmolol had a negative effect on the macrocirculation and failed to improve the affected microcirculation. These negative effects could be explained by a decrease in RV function. First, the observed reduction in RV diastolic function (tau) would have caused a negative effect on preload. This RV dysfunction was confirmed by a markedly elevated CVP, which is associated with reduced microvascular perfusion, increased organ dysfunction and mortality (Damman et al. 2009). Increased tau, after esmolol infusion in a nonseptic setting, has been described in other studies in both the right (Sun et al. 2006) and left ventricle (Firstenberg et al. 2001). This is caused by the negative lusitropic effects of esmolol and is reported to be even more pronounced in the RV compared to the LV (Cortina et al. 2007). Second, the reduced RV dP/dt/P_max reflects an intrinsic myocardial effect and is indicative of an impaired systolic function (Kanzaki et al. 2002; Leeuwenburgh et al. 2002). Esmolol is known for shifting the systolic portion of the pressure–volume downward (Dickstein et al. 1995).

The CVP decreased and the reduced RV systolic and diastolic function recovered after stopping the esmolol infusion. This indicates that esmolol forced the RV to underperform, despites severe hypotension, resulting in low CO. The reversible underperformance was supported by measurements using the single beat method. The P_max_ was unaffected, but the gap between the maximum isovolumetric pressure and end‐systolic pressure became wider. We are the first to report these P_max_ and Pes values of the RV in endotoxic shock to our knowledge. Using these pressures, we showed that the ventricular–arterial coupling ratio (VACR) – as a relatively load independent measure of RV chamber performance – recovered during esmolol infusion. Esmolol improving cardiac mechanical efficiency with reduced myocardial oxygen consumption is in agreement with other studies (Morelli et al. 2016; Du et al. 2017). However, in our model, the observed damped RV systolic and diastolic function outweighs this positive effect. The reduced RV function resulted in a declined MAP to critical values and venous congestion during esmolol infusion. Lowering VACR – in order to increase cardiac efficiency – is only beneficial when adequate perfusion pressures are maintained. This advocates for a more subtle use of esmolol during septic shock.

Here, the microcirculation remained compromised while the perfusion pressure was reduced. This may possibly explain why Jacquet‐Lagreze et al. did demonstrate a small improvement of some of the microcirculatory parameters, since they had an increase in perfusion pressure (Jacquet‐Lagrèze et al. 2015). Our poor microcirculatory state was highlighted by increased lactate levels, microvascular shunting and reduced microcirculatory perfusion parameters. While lactate levels increased significantly, the pCO_2_ gap (Pcv‐aCO_2_) remained stable (<6 mmHg), indicative of microvascular shunting (Haase and Perner 2011). This is supported by the high levels of ScvO_2_ (>85%).

Our data differ from several clinical studies that show beneficial effects of esmolol in patients with septic shock (Morelli et al. 2013a, 2013b, 2014, 2016; Guarracino et al. 2014; Du et al. 2016). These studies show increased SV, maintained MAP and reduced nor‐epinephrine requirements (Morelli et al. 2013a, 2014, 2016). Furthermore, they observe an increased microcirculatory blood flow and improved RV systolic function after esmolol infusion, while the effect in RV diastolic function remained unstudied (Morelli et al. 2013b; Du et al. 2016). These discrepancies with our results could be explained by several factors:

First, a nonlinear dose response of esmolol on MAP, CO, and SV has been reported (Suzuki et al. 2005). Titrating esmolol to a relative opposed to an absolute HR reduction may have required a higher esmolol infusion rate and therefore induce a different hemodynamic response. Therefore, while HR reduction was the primary target, one should never stop assessing other hemodynamic parameters while titrating esmolol.

Second, we only studied the short‐term effects of esmolol. An immediate esmolol induced depression of LV ejection fraction (EF) on day 1 due to the direct negative inotropic effect has been reported, while long‐term effects showed an increase in the EF (Hall et al. 1995). The potent myocardial protection of esmolol by reducing myocardial oxygen metabolism could be more relevant beyond our studied‐acute setting, since endothelial damage would typically occur only after 48 h in clinical setting (Hein et al. 2005). In very early septic shock, reducing HR might counteract the compensatory tachycardia due to a reduced contractility, making this therapy strategy harmful in this condition.

Third, there is no consistency in the (simultaneous) use of fluids and/or vasoactive agents during esmolol therapy, making it hard to compare clinical and experimental results. We kept dobutamine infusion rate constant in all animals but did increase nor‐adrenaline in order to sustain the MAP. Esmolol might have blunted the dobutamine effects, further worsening the right ventricular function.

Last, while high doses of esmolol have consistently been associated with nondeleterious effects on cardiac function in small‐animal models of sepsis (Suzuki et al. 2005; Mori et al. 2011), the results are the opposite in some large‐animal models like ours (Aboab et al. 2011; Jacquet‐Lagrèze et al. 2015). The applied LPS dose and duration vary significantly between different animal models; hence the induced changes and effect of esmolol also vary (Fink 2014). Our loading dose of LPS could have induced a more pronounced cytokine response compared to a continuous infusion only.

### Limitations

We used an experimental animal model with a limited number of animals, only females, and no sham treatment. Stopping esmolol infusion before the end of the experiment allowed us not to use a sham group since the animals were their own controls. However, the effect of the ongoing septic shock and resuscitation maneuvers should be kept in mind. In order to monitor urine production by urethral catheterization, we were limited to using only female animals. Knowing that the ideal model of sepsis does not exist, our sheep model has proven to be convenient, reproducible, representative of the human condition, and met all the criteria of hyperdynamic shock (Nemzek et al. 2008; Kiers et al. 2017).

Furthermore, the duration of the experiment was relatively short [range: 7–9 h], making this experimental design inapplicable to study organ dysfunction and outcome. The duration of each phase could also limit the studied changes in microcirculatory parameters. While the blood flow in the microcirculation can fall quickly, recruitment might have taken place if therapy was prolonged. To further study the effect of HR reduction on the VACR, simultaneous recording of intraventricular pressure and volume is advised. This would allow for assessment of end‐systolic ventricular elastance in order to quantify the ratio between ventricular and arterial elastance. However, we were unable to generate useful ultrasound images of the RV nor absolute end‐diastolic and end‐systolic RV volumes using a conductance catheter in our animal model (McCabe et al. 2014).

In conclusion, in this animal model of acute resuscitated severe endotoxic septic shock, esmolol decreased RV function resulting in reduced perfusion pressure, venous congestion, and unimproved microcirculation. For this reason, clinical diligence and caution are necessary when treating septic shock with esmolol in the acute phase.

## Conflict of Interest

None declared.
